# BioKC: a collaborative platform for curation and annotation of molecular interactions

**DOI:** 10.1093/database/baae013

**Published:** 2024-03-27

**Authors:** Carlos Vega, Marek Ostaszewski, Valentin Grouès, Reinhard Schneider, Venkata Satagopam

**Affiliations:** Luxembourg Centre for Systems Biomedicine, Université du Luxembourg, 7 Avenue des Hauts Fourneaux, Esch-sur-Alzette 4362, Luxembourg; Luxembourg Centre for Systems Biomedicine, Université du Luxembourg, 7 Avenue des Hauts Fourneaux, Esch-sur-Alzette 4362, Luxembourg; Luxembourg Centre for Systems Biomedicine, Université du Luxembourg, 7 Avenue des Hauts Fourneaux, Esch-sur-Alzette 4362, Luxembourg; Luxembourg Centre for Systems Biomedicine, Université du Luxembourg, 7 Avenue des Hauts Fourneaux, Esch-sur-Alzette 4362, Luxembourg; Luxembourg Centre for Systems Biomedicine, Université du Luxembourg, 7 Avenue des Hauts Fourneaux, Esch-sur-Alzette 4362, Luxembourg

## Abstract

Curation of biomedical knowledge into systems biology diagrammatic or computational models is essential for studying complex biological processes. However, systems-level curation is a laborious manual process, especially when facing ever-increasing growth of domain literature. New findings demonstrating elaborate relationships between multiple molecules, pathways and cells have to be represented in a format suitable for systems biology applications. Importantly, curation should capture the complexity of molecular interactions in such a format together with annotations of the involved elements and support stable identifiers and versioning. This challenge calls for novel collaborative tools and platforms allowing to improve the quality and the output of the curation process. In particular, community-based curation, an important source of curated knowledge, requires support in role management, reviewing features and versioning. Here, we present Biological Knowledge Curation (BioKC), a web-based collaborative platform for the curation and annotation of biomedical knowledge following the standard data model from Systems Biology Markup Language (SBML). BioKC offers a graphical user interface for curation of complex molecular interactions and their annotation with stable identifiers and supporting sentences. With the support of collaborative curation and review, it allows to construct building blocks for systems biology diagrams and computational models. These building blocks can be published under stable identifiers and versioned and used as annotations, supporting knowledge building for modelling activities.

## Introduction

**Figure 1.**
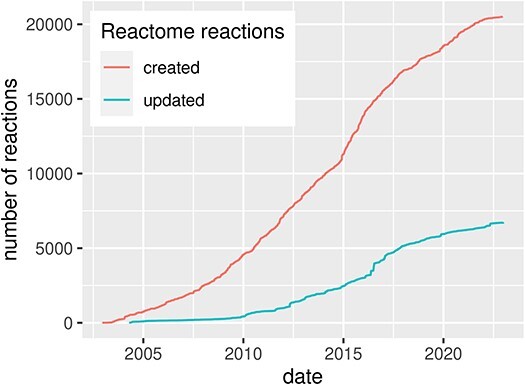
Evolution of the Reactome pathway database, illustrated by the number of created and updated reactions over time, for human pathways. Source: reactome.org  (4).

Since the beginning of computational systems biology during the analogue computer era (2, 3), researchers aim to formalize biological processes into computational models for their analysis and simulations. Diagrammatic representation is an important step of this process, providing a conceptual overview of the formalized knowledge. Usually, knowledge used for building such diagrams is grounded in the existing literature and is extracted and formalized in a process called curation. However, curation in systems biology is time-consuming, requires domain knowledge to explore, organize and encode the information available in the literature, and often involves domain experts to guide and review the process. Despite the challenge, the amount of systems biology diagrams describing molecular mechanisms of health and disease is continuously growing (Figure 1).

Importantly, studying complex molecular processes requires combining multiple literature sources supporting different interactions. For instance, molecular diagrams in review articles are often supported by an extensive body of literature. Similarly, a systems biology diagram is frequently constructed based on multiple pieces of literature evidence and composed of connected building blocks.

Building a systems biology diagram involves (i) extraction of elements, their relationships and annotations from the literature; (ii) construction of diagram following systems biology graphical and modelling notations; and (iii) review and annotation with stable identifiers and literature evidence. A number of pathway databases (4–6) store such diagrams, constructed using different system biology formats and graphical representations and dedicated tools. Importantly, systems biology diagram editors, like CellDesigner (7) or Newt (8) aggregate functionalities of model building (formalization), layout (visual structure, aesthetics) and annotation (literature evidence and stable identifiers). In this setup, biocurators are bound to define aesthetics or details of a diagram layout, which are specifically defined in a given diagram editor. Also, introducing literature annotations to elements or interactions is time-consuming and error-prone, especially when an interaction is supported by multiple literature evidences. Finally, none of the widely used diagram editors support introduction of sentences. This in turn hinders reusability, extension and management (9) of systems biology diagrams, in particular of the annotations and provenance tracking of the literature supporting individual interactions. Moreover, molecular interactions that are common across different cellular pathways need to be either copied across multiple diagrams together with their references or encoded anew if the biocurator is unaware of existing, similar building blocks.

**Figure 2.**
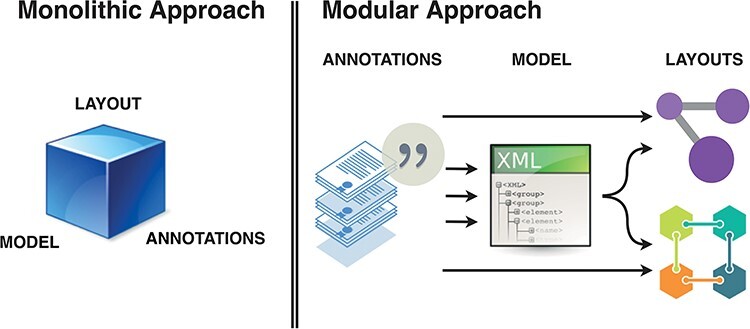
Monolithic vs modular approach to systems biology diagram building. Separation of roles and functions requires interoperability, but offers efficiency of dedicated tools. BioKC supports the first step and modelling interoperability—curation of annotated content compatible with SBML.

Thus, key components of the diagram curation workflow should consider: (i) annotated elements and interactions; (ii) are formalized into modelling building blocks; (iii) which then can be given layout in one or more diagrams. In this ecosystem, a curation platform should allow encoding of biomedical knowledge from literature in a systems biology formalism, and provide stable, versioned annotations. The role of a curator is to create high-granularity, annotated building blocks that can be used in diagram building and referenced as supporting evidence for relevant interactions. Such a modular ecosystem using curated, versioned and identifiable content requires better tool interoperability and collaboration between layout editors, biocurators, annotators and the research community in general (Figure 2).

Here, we focus on curation of such building blocks, for short called fact. For the purpose of this work, we define a systems biology fact as a collection of elements connected by one or more interactions, focused on and representing a molecular mechanism in a systems biology formalism, with associated evidence from the literature. This notion is similar to the curated content of interaction databases (10, 11), as they represent molecular interactions following a defined format and supported by the evidence provided by the curator.

The concept of a fact, understood as a minimal piece of representative knowledge, can be found under various names in the literature. For example, Nano Publications (12) employs a fine-grained model, where a fact, called statement, consists of three basic elements: an assertion, its provenance and publication information. In BioNotate, facts are named ‘snippets’, defined as ‘small chunks of text that may confirm or rule-out a relationship between two known entities’ (13).

Hence, a fact is a piece of knowledge that can be cited, referenced and attributed. In systems biology, a fact also needs to be serialized to a common format, e.g. Systems Biology Markup Language (SBML) and Resource Description Framework (14), using ontologies for term normalization in order to enable interoperable model building. Because a model usually represents multiple interactions of different components, it may consist of one or multiple fact.

In this paper, we introduce Biological Knowledge Curation (BioKC) (15) (available at https://biokb.lcsb.uni.lu), a web-based collaborative tool for fact building, annotation and review. BioKC allows recording annotation and evidence sentences, storing them in a SBML-compliant data model, enabling the user to decide the granularity of their facts. BioKC provides a systematic workflow to ensure high-quality control of the curation process. Once a fact reaches maturity, it can be released with a stable uniform resource identifier, supported by Identifiers.org registry (see https://registry.identifiers.org/registry/biokc). Such a fact can then be referenced in systems biology editors as an annotation to an interaction or by other tools.

BioKC is built on top of BioKB (16), a web-based interface designed to browse the text-mining results of almost 3 million biomedical publications including both abstracts and full-text articles. BioKC is a novel platform that enables the user to construct building blocks of systems biology models and allows the user to annotate them with human-provided and machine-identified literature evidence. Knowledge representation in BioKC follows the SBML standard in formalizing elements of a given model, their relationships and annotations.

In the following sections, we briefly review the current state of the art and approaches for systems biology curation and annotation. Next, we describe the functionalities and design of BioKC supported by use cases. Finally, we describe further steps foreseen in the development of the platform.

## Related work

Many tools for annotation and curation of biomedical publications, despite being described with similar keywords, showcase a broad range of features and purposes. In general, these tools are either suited for the annotation and curation of publications or focused on visual model building.

### Annotation and curation of publications

Different tools for knowledge curation were reviewed in two thorough surveys and evaluated using detailed criteria. The first review is specifically devoted to biomedical literature annotation tools, featuring 35 criteria used to evaluate 13 annotation tools (17). Although this survey makes a distinction between text annotation (i.e. a complete tagging of a given text) and text curation (i.e. document analysis with respect to a given context), it does not consider model curation from the annotated/curated text information as a feature. Most of reviewed tools are no longer available or do not feature collaborative web functionalities.

A recent survey from the same authors (18) covers a higher number of annotation tools but not all of them are directly related to the biomedical sciences (18). In this case, 78 tools were selected following 26 criteria, and 15 tools were evaluated in detail. Most of the discarded tools were not available or were not web based. Finally, only BioQRator (19), ezTag (20), MyMiner (21) and tagtog (22) were suitable for biomedicine. However, none of these four tools support model curation or systems biology formats.

Table 2 summarizes the criteria used in (18) except the publication impact of the tools, as these are not relevant for this work. Importantly, the criteria from Table 2 will be used for the description and evaluation of our tool, BioKC, in ‘Technical and Functional Comparison’ section. We extended the table by including the tools relevant for curation in systems biology and biomedicine. In particular, we consider systems biology diagram editors and viewers as tools for curation because of their capability for model building and review.

**Table 2.** Technical, data-related and functional criteria) (18)

Technical criteria	Data criteria	Functional criteria	
T1—Date of the last version	D1—Format of the schema	F1—Allowance of multi-label annotations	F8—Allowance for saving documents partially
T2—Availability of the source code	D2—Input format for documents	F2—Allowance of document-level annotations	F9—Ability to highlight parts of the text
T3—Online availability for use	D3—Output format for annotations	F3—Support for annotation of relationships	F10—Support for users and teams
T4—Easiness of installation		F4—Support for ontologies and terminologies	F11—Support for inter-annotator agreement
T5—Quality of the documentation		F5—Support for pre-annotations	F12—Data privacy
T6—Type of license		F6—Integration with PubMed	F13—Support for various languages
T7—Free of charge		F7—Suitability for full texts	

Publication criteria have been excluded as they do not apply for the comparison conducted in this paper.

### Editing and visualisation of curated knowledge

Many existing tools are able to create and parse systems biology diagrams encoded in different formats (e.g. SBGN-ML, SBML with ‘layout’ extension) allowing the user to curate a layout or annotate a model. The authors in (9) present a comparison of software tools suited to work with diagram layouts in systems biology standard formats. They differentiate between ‘diagram editors’, like CellDesigner (7, 23), Newt (8) or Cytoscape (24), and ‘management platforms’ which include pathway databases as Reactome (4), Kyoto Encyclopedia of Genes and Genomes (5) or WikiPathways (6), and platforms for visualization of contextualised networks like MINERVA (25), NaviCell (26) or BioUML (27). An important drawback of diagram-based model building is annotating the content. Diagram editors have limited capability to provide supporting evidence. Another notable example is the CellCollective platform for visually aided construction of Boolean models (28), providing user interface to construct models online and annotate them. However, standardized annotations (29) are difficult to introduce and maintain in these tools despite support by modelling languages and functionalities implemented to handle them.

Despite long-standing development of diagram editors and pathway databases, the reuse of building blocks repeating across diagrams is not well addressed to date. Systems Biology Graphical Notation (SBGN) Bricks (30) is an important effort in this direction, defining graphically recurring motifs in systems biology diagrams. Supported by Newt Editor (8), it facilitates harmonized diagram construction. Nevertheless, the annotation part of such blocks is missing, leaving up to the diagram author to supply all necessary annotations and supporting evidence. In this context, Reactome curation stands out as interaction centric, with dedicated identifiers, annotations and curation log (4).

### Summary

The ecosystem of tools for systems biology curation (Table 1) offers solutions for publication annotation, layout editing and knowledge exploration. Nevertheless, there is a lack of platforms for quality-controlled model curation allowing online collaborative work. Some publication annotation tools like BioQRator support collaborative curation but do not offer model building features. ‘Web repositories’ and databases like BioModels (34) and CIDeR (35) host a multitude of models that can be downloaded in SBML, but these are ‘read-only’ services. Interestingly, PathText2 (36) is a step in the right direction, as it was designed to annotate biological pathway models with supporting knowledge from the literature, using SBML contents to query multiple databases and text-mining services. Similarly, CellCollective (28) implements some of the necessary functionalities for reproducible model construction, where components and interactions can be annotated with text notes.

**Table 1.** Summary depicting purpose, online availability and capabilities of different tools

Purpose	Tool	Browser tool	Online tool	Collaborative	Linkable	Annotation	Layout aware	SBML SBGN
General text annotation	tagtog (22)	✓	✓	✓	✓	✓	✗	✗
	brat (31)	✓	✗	✓	✗	✓	✗	✗
	WebAnno (32)	✓	✗	✓	✗	✓	✗	✗
	FLAT (33)	✓	✗	✓	✗	✓	✗	✗
Biomedical text annotation	MyMiner (21)	✓	✓	✗	✗	✓	✗	✗
	BioQRator (19)	✓	✓	✗	✓	✓	✗	✗
	ezTag (20)	✓	✓	✗	✓	✓	✗	✗
Diagram editor	CellDesigner (23)	✗	✗	✗	✗	✓	✓	✓
	Newt (8)	✓	✓	✗	✗	✓	✓	✓
Visual repository	WikiPathways (6)	✓	✓	✓	✓	✗	✓	✗
	KEGG (5)	✓	✓	✗	✓	✗	✓	✗
	Reactome (4)	✓	✓	✗	✓	✗	✓	✗
	CellCollective (28)	✓	✓	✓	✓	✓	✗	✗
Visualization platform	Cytoscape (24)	✗	✗	✗	✗	✓	✓	✗
	NaviCell (26)	✓	✓	✗	✓	✓	✓	✓
	MINERVA (25)	✓	✓	✗	✓	✓	✓	✓
	BioUML (27)	✓	✓	✓	✗	✓	✓	✓

Some browser-based tools are not available online, and this distinction is shown in the first two columns. ‘Collaborative’ column states which tools allow multi-user simultaneous operation. ‘Linkable’ criterion refers to the ability to share and use the tool output as annotable content via uniform resource identifier-like links. Conversely, the ‘Annotation’ criterion indicates if a tool is able to produce annotations on the content.

Importantly, tools with graphical user interface like diagram editors or publication annotation tools are not the best suited for high-quality curation, as they have limited capabilities to support in-depth curation, e.g. handling supporting sentences from scientific articles or versioning particular interactions. On the other hand, tools relying on text interfaces and representation formats allow for detailed annotation but offer limited functional interfaces to assist curation. There can be many possible reasons for this situation, including (i) limited resources for tool development, where functionalities of diagram building and annotation cannot be covered in depth; (ii) focus on a particular use case or user group, e.g. diagram designers or biocurators; or (iii) evolution of user needs and workflows, including increasingly collaborative and interdisciplinary research community.

Considering the above, the motivation for BioKC is 3-fold. First, we aim to provide a web application for collaborative curation and annotation of systems biology building blocks. Second, we want to implement features for a systematic curation workflow that will facilitate knowledge building and increase quality control. Finally, we seek to decouple the tasks of knowledge curation and of diagram building in systems biology. In this scenario, curated and reviewed building blocks—facts—having stable identifiers can be used to annotate relevant interactions in systems biology diagrams.

## Results

### Features

#### Structure of a fact

In BioKC, a fact follows the SBML notation (1) and features multi-element interactions (SBML’s reactants, products and modifiers), enclosed in compartments. All components of a fact can be annotated with stable identifiers from the Identifiers.org registry (37). Moreover, users can control the visibility of facts they curate, organize them in groups, assign tasks and release them in a version controlled manner. BioKC allows editing facts and the properties of their elements via web interface, adding more components or annotations and maintaining a change log that records such actions (Figure 4).

#### Annotation of a fact

All SBML elements inside a fact can be annotated with stable identifiers supported by Identifiers.org registry (37) having over 800 different namespaces and defined using BioModels qualifiers (https://co.mbine.org/standards/qualifiers). Such elements can also be annotated with supporting evidence from the literature either from BioKB or third-party sources. Sentences from third-party sources can be imported via both the basket and the fact curation interface (Figure 4b and c). Basket mode supports bulk import of sentences from tsv files. Conversely, single sentences from third-party sources can be added via the fact curation interface. In both cases, provenance information can be provided, and a valid Digital Object Identifier, PubMed Central or PubMed ID allows to retrieve the corresponding publication metadata for the annotation.

#### Fact groups and multi-user workflow

For a flexible management of the facts, they can be gathered in groups with private or public visibility. Users may be members of multiple groups and have different roles in each group. Group managers can grant read, annotation, curation or management permissions to other members of the group. A warning will be shown if a user tries to curate or annotate a fact, while another user is working on it.

#### Role system

BioKC users can have different roles, assigned per group of facts. These roles correspond to specific sets of permitted actions. ‘Managers’ can administer their groups and member permissions, delete facts or decide about the completeness of a task. ‘Curators’ are able to add or edit elements composing a fact, creating and defining its structure. ‘Annotators’ cannot edit a structure of a fact but can assign annotations and sentences to its elements, completing its evidence. Finally, ‘Readers’ can inspect facts but cannot modify any aspect of them.

### Usage

**Figure 3.**
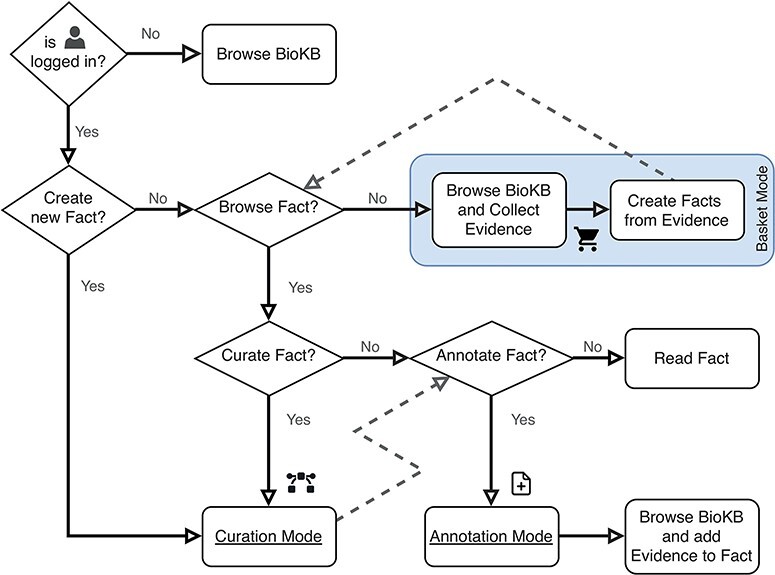
Flowchart describing the user operation flow and the different operation modes. The box shows the basket mode, which is the default operation mode when both curation and annotation modes are disabled.

**Figure 4.**
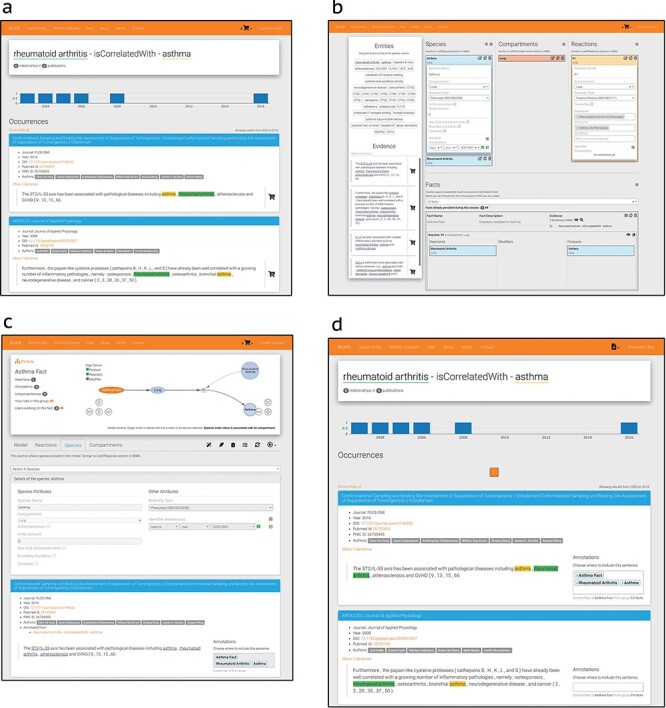
BioKC interface and functionalities. (**a**) BioKB relationship view showing sentences for a given entity relationship, and the sentences can be added to the basket. (**b**) Basket checkout redirects to the basket view where facts and their elements can be composed. (**c**) The ‘Fact view’ is where facts can be edited, either from scratch or after being persisted in the basket view. (**d**) The annotation mode enables annotation capabilities in BioKB to assign supporting evidence to one or multiple elements of a fact.

BioKC supports two curation workflows: (i) by first collecting the evidence, then creating facts from it (see ‘Basket Mode’ box in Figure 3) or (ii) by starting with the curation of a fact and then annotating it with supporting evidence. Moreover, a fact can be directly imported from an SBML file, having all its elements, interactions, compartments and annotations stored in BioKC. This allows harmonization across SBML-compatible sources (9), further annotation and versioning of individual interactions. Note the icon 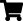 on the top right corner in Figure 4a and b. This corner indicates the current operation mode.

The default is the ‘basket mode’ for collecting evidence—the first workflow. The evidence can be collected from BioKB using the the icon 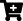 on the sentences (Figure 4a). This icon is activated when using BioKC, enabling users to collect evidence while browsing BioKB. Alternatively, a set of evidences can be supplied externally using a tsv file. Such sentences collected in the ‘Basket view’ can be then used to construct one or multiple facts (Figure 4b). Once these facts are persisted, they can be further edited in the ‘Fact view’ (Figure 4c).

The second workflow is depicted in Figure 4c and d. It involves creating the enclosing compartment, defining building blocks of a fact and connecting them with interactions. During this process, a curator annotates elements, interactions and the entire fact and provides literature evidence.

#### Curation

Users with curation permissions can start the ‘curation mode’ from the ‘Fact view’ (see 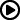 in Figure 4c) to add, delete or edit the elements that compose a fact and annotate it using resolvable identifiers. Top right corner will show the icon 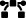 indicating that the curation mode is enabled.

#### Annotation

Similarly, annotators can start the ‘annotation mode’ to add or remove supporting evidence from a fact. Such sentence annotations can be assigned to one or multiple parts of a fact, including the root element. Image (d) in Figure 4 shows how sentences in BioKB include a select box while the annotation mode is enabled. This mode also enables the annotator box (see icon 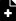 in Figure 4d) which lists recently visited pages. Sentences from external sources can be imported from the fact view using the ‘custom sentence annotation tool’ (see 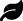 in Figure 4c).

#### Example use scenarios

The above-mentioned functionalities make BioKC a useful solution in a number of possible scenarios. The first is a typical biocuration of domain-specific literature into interactions based on selected articles, where a curator extracts individual interactions from a corpus of pre-selected papers to construct a set of reusable interactions, similarly to interaction-based databases like SIGNOR (11). Such interactions can be released as facts, with stable identifiers and versions, reviewed by assigned peers (see ‘Materials and Methods’ section). A result of such a curation can be found here: https://biokb.lcsb.uni.lu/fact/bkc640. Version history and all associated annotations and evidence are accessible via the tabs below the diagrammatic representation of the fact.

The second scenario involves construction of a disease map—a dedicated systems biology repository (38). Disease maps are human- and computer-readable repositories containing manually curated interactions following the SBML format, organized into diagrams according to the SBGN notation. The process of map development (39) involves intensive biocuration work over a selected body of literature. Importantly, interactions of disease maps reference the source literature. BioKC supports this process allowing to construct a fact containing multiple literature references and cited sentences in a versioned and reviewed manner and then use the fact identifier as a sole reference in the diagram interaction.

The final scenario involves curation based on large lists of content from text mining, where curator’s attention is required to refine already pre-formated content. In this scenario, contents of the text mining are uploaded to BioKC for quick construction of facts and for persisting them for downstream use. This workflow was, for instance, applied in the COVID-19 Disease Map project (40). There, BioKC was used as a tool for triage and SBML formatting of interactions extracted from the LitCovid corpus (https://www.ncbi.nlm.nih.gov/research/coronavirus/) for curation of systems biology diagrams.

#### Review

**Figure 5.**
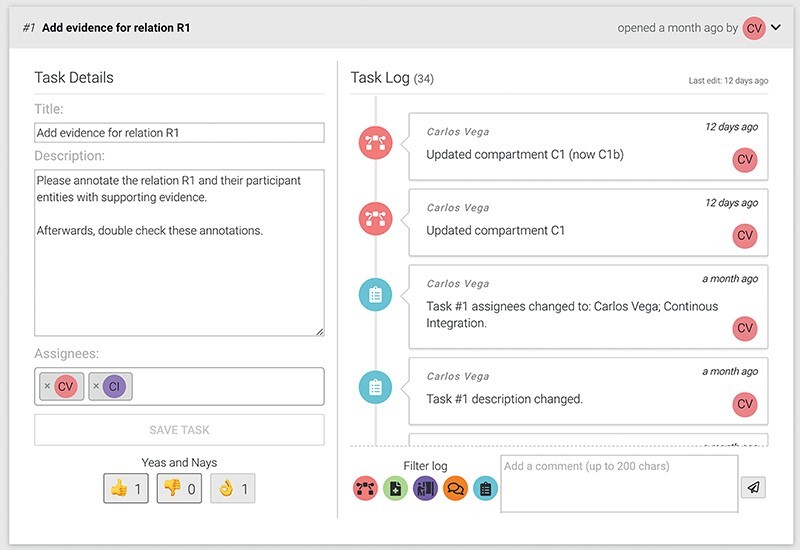
An example of a task showing the title, description, assigned users and cast votes on the left. The right side shows the task log and the comment input box.

BioKC provides quality control and review mechanisms for the curation and annotation of facts. In particular, group managers can assign tasks to users from the ‘Fact view’ (see icon 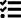 in Figure 4c) to guide the curation and annotation of the fact. An annotator agreement system allows users to assess the task progress by exchanging messages and casting votes regarding their agreement or disagreement with the task completion. Managers have the final word over the task completion casting the ‘mark as finished’ vote (see the ‘ok hand’ icon in Figure 5). Once certain positive quorum is met, the task is marked as completed. This functionality allows to address conflicting evidence relevant for a given fact. However, as BioKC is a distributed curation system, it does not check for potential conflicts between independently curated facts.

## Discussion

High-quality curation is key to provide reliable systems biology building blocks. User-friendly annotation, collaborative features and quality control mechanisms are essential for such a task. BioKC facilitates the process of curating annotated molecular interactions in a standard and interoperable format as SBML, allowing their later use in diagram or model building.

Table 1 showcases the tools offering curation capabilities. Although most are web-based, collaborative features are offered by only a few, mainly text annotation tools. BioKC was designed bearing in mind many capabilities from the diverse range of available tools, particularly those closely related to text annotation tools. We present a detailed comparison of text annotation aspects later.

### Technical and functional comparison

Here, we use the criteria from (18) to compare technical, data-related and functional aspects of BioKC and other tools. In the original evaluation, points were assigned for completely (1), partially (0.5) or not (0) fulfilling a criterion. The sum of points was divided by the number of criteria, with a maximum score of 1. In the evaluation, tools obtained an average score of 0.62. Three best tools were WebAnno (32) (0.81), brat (31) (0.75) and FLAT (33) (0.71). Besides, a dedicated section was included for tools suitable for biomedicine, including ezTag (20) (0.67), BioQRator (19) (0.58), tagtog (22) (0.6) and MyMiner (21) (0.52).

We have included these seven tools in our comparison and recalculated the scores excluding the criteria not applicable for this paper: year of last publication (P1), citations in Google Scholar (P2) and citations for corpus development (P3). The results can be found in Table 3, showing that BioKC coverage of the evaluation criteria is higher than other annotation tools, including those suitable for biomedicine.

**Table 3.** The criteria for comparison of technical and functional aspects following the criteria from Table 2

Tools	Technical							Data			Functional													Score				
	T1	T2	T3	T4	T5	T6	T7	D1	D2	D3	F1	F2	F3	F4	F5	F6	F7	F8	F9	F10	F11	F12	F13	T	D	F	Total	Score
**BioKC**	✓	✓	✓	✓	✓	✓	✓	✓	✓	✓	✓	✓	✓	✓		✓	✓	✓	✓	✓	*	*		7	3	10	20	**0.87**
FLAT	✓	✓	✓	✓	✓	✓	✓	✓	✓	✓	✓			✓	*		✓	✓	✓	✓		✓	✓	7	3	8.5	18.5	0.8
WebAnno	✓	✓			✓	✓	✓	✓	✓	✓	✓		✓	✓	*		✓	✓	✓	*	✓	✓	✓	5	3	10	18	0.78
brat	*	✓		*	✓	✓	✓	*	✓	*	✓		✓	✓	*		✓	✓	✓	*	*	✓	✓	5	2	9.5	16.5	0.72
Tagtog			*	✓	✓		*	✓	✓	✓		✓	✓	✓		✓	✓	✓	✓				✓	3	3	8	14	0.61
ezTag	✓	✓	✓	✓	✓		✓	✓	✓	✓				✓	✓	✓	✓	✓	✓	*		✓		6	3	7.5	16.5	0.72
BioQRator	*		✓	✓	✓		✓	✓	✓	✓			✓	✓	*	✓		✓	✓	*				4.5	3	6	13.5	0.59
MyMiner			✓	✓	✓		✓	✓	*	*		✓	*		*	✓		*	✓		*		✓	4	2	6	12	0.52

The ‘✓’ symbol indicates total fulfilment, ‘*’partial fulfilment and empty cells mean no fulfilment. Tools are sorted by their descending score. Publication annotation tools suitable for biomedicine are BioKC, Tagtog, exTag, BioQRator and MyMiner.

Nevertheless, some criteria for BioKC are either partially fulfilled (F11, F12) or not fulfilled at all (F5, F13). The F11 criterion is partially satisfied, since even though BioKC provides mechanisms to ensure certain level of inter-annotator agreement, it does not entail a fully blind annotation and curation workflow. Similarly, F12 criterion can be fulfilled as long as curated facts are kept private to their group members but not once a fact is publicly released. F13 criterion is not satisfied since the platform dictionaries are in English. Lastly, F5 criterion is not met since annotation import is not supported.

In summary, BioKC covers all technical and data criteria and most of the functional aspects of text annotation tools in (18). Notwithstanding, this evaluation compares tools regarding their annotation capabilities, while their main purpose differs from the aims of BioKC. Consequently, these criteria are not entirely exhaustive as some capabilities offered by BioKC are not covered. Such capabilities have been described separately in previous sections (see ‘Features’ section).

### Curation guidelines compliance

To complete the assessment of BioKC, we referred to a recent work from (41) introducing the Minimum Information about a Molecular Interaction CAusal STatement (MI2CAST). MI2CAST consists of rules and good practices for the curation of causal molecular interactions. The first three rules cover mandatory information about the interaction: (i) the source and target entities, (ii) the effect of the interaction, and (iii) the evidence provenance. Additionally, the fourth rule recommends encoding contextual information.

MI2CAST guidelines do not impose a particular format in which interactions should be represented or encoded. Nevertheless, BioKC encodes interaction elements following SBML as reactants, products and modifiers, supports type specification with Systems Biology Ontology, satisfying first two rules. Also, it enables annotation of all components of a given fact and the fact itself with publication identifiers and with the sentence itself, satisfying latter two rules. Therefore, we strongly believe that features and capabilities of BioKC described in this paper comply with MI2CAST guidelines and recommendations.

## Conclusions

We present BioKC, a web-based platform for collaborative curation and annotation to cope with the new needs of curation for systems biology. Our platform offers quality control and reviewing features for curation and annotation that are not available in the current state of the art. These include (i) systems biology-focused curation of molecular interactions, compliant with SBML standard; (ii) annotation of facts with sentences and references to literature and bioinformatic databases; and iii) collaborative curation setup, allowing different roles, inter-curator agreement publishing and versioning of curated facts.

BioKC platform is in constant development and its roadmap (https://biokc.pages.uni.lu/roadmap/) foresees support for defining and annotating complexes and handling of SBML extensions such as the Multistate and Multicomponent species package (42). Supporting a wider range of text-mining knowledge bases and modelling formats and repositories is essential for further interoperability. With our work, we aim to ease research collaboration providing features to review the curation process and to perform quality control of the annotation of supporting evidence.

## Materials and methods

### Architecture

The proposed solution, BioKC, extends BioKB functionality. BioKB is a platform designed to help researchers easily access semantic content of millions of abstracts and full-text articles (16). BioKB relies on the INDRA text-mining pipeline (43) that extracts relations between a wide variety of concepts, including proteins, chemicals, diseases, biological processes and molecular functions, encoded as causal interactions. Extracted knowledge is stored in a knowledge base publicly accessible via a web interface (BioKB) and a SPARQL endpoint.

#### Implementation Environment

BioKC is developed in Python and JavaScript, which allow for fast iterative development life cycle in both front end and back end. Flask and SQLalchemy are employed for the web server and database implementations, with Vue, Jquery and other JavaScript libraries contributing to a real-time collaborative and interactive multi-user experience in the client side.

#### Multi-level annotation

**Figure 6.**
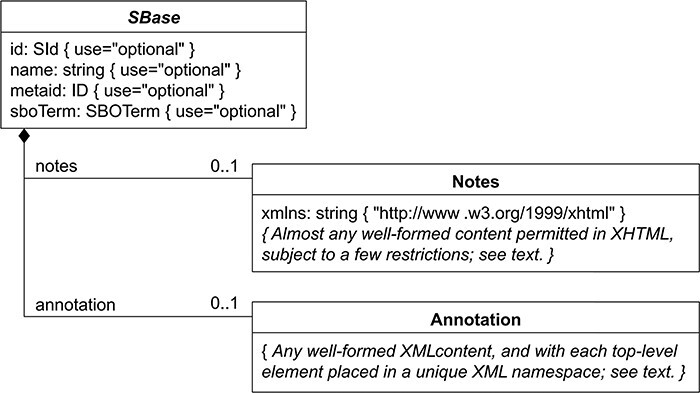
Nearly every object composing an SBML Level 3 model definition has a specific data type that is derived directly or indirectly from a single abstract type called SBase. See Section 3.2 from SBML Specification for Level 3 Version 2 Core. BioKC follows the same structure for all SBML elements composing a fact so that they can be annotated.

BioKC follows a SBML-like data model in which every object inherits all properties from SBase abstract type depicted in Figure 6. This hierarchy was replicated using SQL joined table inheritance polymorphism. Hence, data model tables like Species, Compartment, Reaction, SpeciesReference, etc. inherit these properties allowing annotation at different levels (i.e. annotations can be assigned to compartments, elements, etc.).

#### Versioning and stable identifiers

Curators can release stable versions of a given fact. A stable identifier is then assigned to the fact, within the namespace registered in the Identifiers.org registry (https://registry.identifiers.org/registry/biokc). The contents of such released fact are stored in a dedicated database. Each released version has its contents registered as a persistent record. The most recent version of a fact is stored under its base identifier (e.g. https://biokb.lcsb.uni.lu/fact/bkc639), but earlier versions are available as well (e.g. https://biokb.lcsb.uni.lu/fact/bkc639v2). A graph illustrates the version history, together with release notes provided by curators.

#### Action log

Multi-user collaborative work requires registering the actions for a given fact. For this, we employ a custom declarative base class and a SQLalchemy *mixin*, allowing adding common columns to multiple tables that share this functionality. Specifically, each table has two columns, created_on and updated_on, that register the creation date and last modification time, respectively. Benefiting from previously described data model polymorphism, each action is registered in the modified element as a Note (Figure 6) with a User as author and a comment describing the action. Such actions can be assigned to multiple tasks to better organize the actions taken during the curation process.

## Data Availability

The authors state that data sharing is not applicable to this article as no datasets were generated or analysed during the current study.
